# Instruction in Sexual Hygiene

**Published:** 1912-01

**Authors:** 


### Instruction in Sexual Hygiene

DURING the last few days of October, Dr. Winfield Scott Hall of Chicago, gave several popular lectures in Buffalo on this topic. Last winter, Dr. Richard Cabot of Boston, spoke in the city on a somewhat similar subject. Both of these men are far from the ordinary type of professional lecturers or reformers. Rightly or wrongly, the sincerity of one who earns his living by any form of philanthropic or moral work, is subject to question. Rightly or wrongly, the authority of one who essays such work is questioned, if he undertakes it without special technical training. No such criticism can be made, of either Hall or Cabot. Both are men of attainments and high standing in the medical profession, ballasted by serious interest in a life work. They are in no danger of being capsized by a storm of either approval or disapproval or of being driven out of a rational conservative course, as is the professional reformer, empty of actual knowledge guided merely by enthusiasm and liable to be turned by ambition and even self interest without realizing it.

Jane Addams, in the October *McClure,* writes delicately but plainly of the white slave trade and prophecies its abolition, drawing a parallel to the development of the struggle against negro slavery.

These are only a few of many, manifestations of the awakening of the public conscience. The country is becoming less prudish and more moral—perhaps not more moral in the limited sexual sense, and according to actual practice, but, at any rate, more moral in the general sense of demanding fair play, of protecting the weak, of guiding the ignorant, and of insisting on the relief and prevention of suffering and disease, however caused. It is a sign of morality in the broadest sense, that one does not so often hear pious men compare degenerate and normal sexual practices to the advantage of the former; that it is no longer considered undesirable, if not absolutely wicked, to prevent blindness, chronic invalidism, paralysis and the like, on the ground that venereal disease is a proper punishment; that even very good persons are beginning to realize that there is a difference between being bad and being dishonorable, filthy and depraved.

As to whether the country is more moral than formerly, in the limited, sexual sense, it is difficult to say. There are no statistics to guide one and general impressions are apt to be misleading for the mere fact that, the older one grows, the more opportunities he has to acquire facts and the less hesitation have others to confide in him. There is immorality enough in all classes of society, in those where it may be excused by poverty, in those where it may be explained by idleness and the habit of self indulgence fostered by wealth, in those where hard work, physical or intellectual, sobriety, religion and refinement might well lead us to expect the highest standards of sexual ethics. After each discovery that some idol has feet of clay, we tend to become cynic, even to the extent of asking how there can be a white slave trade when there is an abundance of free labor. But, against such cynicism, we must fortify ourselves by remembering that the very reason that such instances impress us so forcibly is that they are the exception.

There are some considerations that must not be overlooked in educating the youth in regard to sexual matters. A very important point is that the purity of the little boy is just as important as that of the little girl. Even if this be not conceded as a general proposition, it needs little thought to show that a boy, led astray, usually becomes a source of evil to the opposite sex. Two of the worst rakes we have known were started on their career by teachers. A high school girl recently boasted to a physician that she belonged to a clique that delighted in seducing green boys.

Another point to be remembered by all lecturers and writers of pamphlets on six topics is that children already have a pretty fair understanding of the subject—derived from older boys, from the surreptitious reading of quack circulars, home medical compends and from that authority which they regard as infallible: the boy that works in the drug store. Many adults fail to realize how much children know and how shrewdly they think.

However great the immediate effect of an exaggerated, insincere talk in which the moral phase of the subject outweighs considerations of physiologic and pathologic fact, the ultimate outcome of a policy of instruction will be to instruct and the discussion, weighing of evidence and seeking for other authority will bring out the absolute truth and discredit any moralist who acts on the supposition that the end justifies the means. It must not be forgotten that any large audience, especially of older boys and girls, will probably include some already well informed, theoretically and practically, and who are looking for "pointers."

One further item must be borne in mind: the right of parents to prefer the old-fashioned policy of carefully guarded innocence. We must confess to a leaning toward this policy and there are certainly instances in which it works well, although, for the most part, it is a parental theory rather than a juvenile fact. However, it is questionable whether information on sexual matters should be forced on the young contrary to the wishes of parents.

